# A Review of Botanical Characteristics, Traditional Usage, Chemical Components, Pharmacological Activities, and Safety of *Pereskia bleo* (Kunth) DC

**DOI:** 10.1155/2014/326107

**Published:** 2014-06-03

**Authors:** Sogand Zareisedehizadeh, Chay-Hoon Tan, Hwee-Ling Koh

**Affiliations:** ^1^Department of Pharmacy, Faculty of Science, National University of Singapore, 18 Science Drive 4, Singapore 117543; ^2^Department of Pharmacology, Yong Loo Lin School of Medicine, National University of Singapore, Singapore 117597

## Abstract

*Pereskia bleo*, a leafy cactus, is a medicinal plant native to West and South America and distributed in tropical and subtropical areas. It is traditionally used as a dietary vegetable, barrier hedge, water purifier, and insect repellant and for maintaining health, detoxification, prevention of cancer, and/or treatment of cancer, hypertension, diabetes, stomach ache, muscle pain, and inflammatory diseases such as dermatitis and rheumatism. The aim of this paper was to provide an up-to-date and comprehensive review of the botanical characteristics, traditional usage, phytochemistry, pharmacological activities, and safety of *P. bleo*. A literature search using MEDLINE (via PubMed), Science direct, Scopus and Google scholar and China Academic Journals Full-Text Database (CNKI) and available eBooks and books in the National University of Singapore libraries in English and Chinese was conducted. The following keywords were used: *Pereskia bleo, Pereskia panamensis, Pereskia corrugata, Rhodocacus corrugatus, Rhodocacus bleo, Cactus panamensis, Cactus bleo*, Spinach cactus, wax rose, Perescia, and Chinese rose. This review revealed the association between the traditional usage of *P. bleo* and reported pharmacological properties in the literature. Further investigation on the pharmacological properties and phytoconstituents of *P. bleo* is warranted to further exploit its potentials as a source of novel therapeutic agents or lead compounds.

## 1. Introduction

*Pereskia bleo* is a medicinal plant of the family Cactaceae. Cacti are well-known desert plants and widely recognized by their specialized growth form of the stems and leaves. This family consists of 100 genera and about 2000 species [1, 2]. The genus* Pereskia* consists of 17 species with regular leaf development and function. They are generally representative of the “ancestral cactus.” This genus does not look much like other types of cacti because of having substantial leaves and thin stems [3–5]. The plants in the genus* Pereskia* originate from the region between Brazil and Mexico and South America and Central America [6–8] and are cultivated in many tropical and subtropical countries including India, Malaysia, Singapore, and Indonesia [1]. They also generally resemble other types of plants such as roses [3, 8].* Pereskia* species are divided into Clades A and B [9] (Table 1). The two clades of* Pereskia* differ in their geographical distribution. Clade A is found around the Gulf of Mexico and the Caribbean Sea whereas Clade B is found in the south of the Amazon Basin. The stems of the species of* Pereskia* within Clade A begin to form bark early in the life of the plant like most non-cacti. In contrast,* Pereskia* species within Clade B typically delay forming bark, thus giving the stem the potential to become a major organ for photosynthesis [4].

Among them,* Pereskia aculeata *Mill (*P. aculeate*),* Pereskia grandifolia* Haw (*P. grandifolia*), and* Pereskia bleo* (Kunth) DC. (*P. bleo*) are listed to be found in Singapore and Malaysia [7, 10, 11].* P. bleo and P. grandifolia *are used for medicinal purposes in these areas [1, 11]. Hence, more information on these three species is presented below.

### 1.1. *Pereskia aculeata* Mill

Its common names are Barbados gooseberry or lemon vine [12, 13] and it is native to tropical America [14]. This plant is a scrambling vine growing to the height of 10 m to a tree. The stems reach 2-3 cm in diameter. Younger stems have hooked thorns and older stems have clusters of woody spines. The leaves are 4–11 cm long and 1.5–4 cm wide, simple, and deciduous in the dry season. The flowers are white, cream, or pinkish with 2.5–5 cm diameter and strongly scented. This plant has translucent rounded white to pink berries which turn to yellow or orange with the diameter of 2 cm upon ripening. The fruits are edible and containing numerous small seeds. They somewhat resemble the gooseberry in appearance and are of excellent flavor [15, 16]. The leaves are also edible and are a popular vegetable in parts of the Brazilian state of Minas Gerais under the name of ora-pro-nóbis [14].

### 1.2. *Pereskia grandifolia* Haw

It is also known as rose cactus or* Rhodocactus grandifolia*. This plant is native to the Northeastern Brazil restingas and is cultivated in tropical and subtropical areas [7]. It is a shrub or small tree, 2–5 m high, with a grayish-brown trunk up to 20 cm in diameter. The spines range from black to brown and their number at each areole gradually increases with age. The new twigs may be spineless while the trunk may have up to 90 spines in areoles, each 2–6.5 cm long. The leaves vary in size from 9 to 23 cm long and the shapes range from elliptic to ovate and obovate-lanceolate. Usually 10–15 flowers of dense inflorescence develop at the ends of stems, but sometimes there are 30 or more. The flowers are pink-purple and look like rose with 3–5 cm diameter [12]. The leaves of* P. grandifolia* are edible [11].

### 1.3. *Pereskia bleo* (Kunth) DC

*P. bleo *is also known as* Cactus bleo* and has been commonly used for a variety of medicinal and non-medicinal purposes in different countries [1, 2]. However, to the best of our knowledge, a comprehensive review of* P. bleo* is not available. The objective of this paper is to provide a comprehensive review of the botanical characteristics, traditional usage, phytoconstituents, pharmacological activities, and safety of* P. bleo*. Such information will serve as a useful resource for the proper usage of this plant and for future research.

## 2. Method

Internet sources including MEDLINE (via Pubmed), Science direct, Scopus and Google scholar, and China Journals Full-Text Database (via CNKI) were searched for publications on this plant. The following keywords were used:* Pereskia bleo, Pereskia panamensis, Pereskia corrugata, Rhodocactus corrugatus, Rhodocactus bleo, Cactus panamensis, Cactus bleo*, Spinach cactus, wax rose, Perescia, and Chinese rose. No restriction on the language and date of publication was implemented. In addition, available books and eBooks in the National University of Singapore (NUS) libraries were manually searched for the relevant information.

## 3. Results and Discussion

### 3.1. Botanical Characteristics

*P. bleo *belongs to the order of* Caryophyllales Juss. ex Bercht. *&* J. Presl*, superorder of* Caryophyllanae Takht* and subclass of* Magnoliidae Novák ex Takht*. It is in the Cactaceae family,* Peresioideae* subfamily, and* Pereskia* Mill genus [44, 45]. In the International Plant Nomenclature Index (IPNI) [46], its ID code is 273592-2 and its basionym is* Cactus bleo *(Kunth). Basionym name is defined as “previously published legitimate name-bringing or epithet-bringing synonym from which a new name is formed for a taxon of different rank or position taxon of different rank or position” [17]. The scientific and common names of* P. bleo* are listed in Table 2. This plant is also known as “Pokok Jarum Tujuh Bilah” in Malay and “Cak Sing Cam” or “Qi Xing Zhen (*七星针*)” in Chinese [8, 40]. Its Chinese name literally means “seven stars needle” [7].

*P. bleo *originates from Mesoamerica (Panama), Western South America (Columbia) [1, 2, 6, 12] and is distributed in tropical and subtropical regions [1, 2]. It is a deciduous, shrubby, tree-like plant with a height of 0.6–8 m. The trunk reaches 10 cm in diameter and bears very large fascicle of spines when it is young. However, the trunk becomes naked when turning old. Young branches are red and leafy and often bear 5–7 black spines up to 1 cm in length. The spines reach 2 cm on the older stems. The leaves are thin, oblong to oblanceolate, glossy, and succulent, 6–21 cm long, and 2–7 cm wide [2]. The flowers are orange-red and grouped in 2–4 terminally and laterally. The fruits are yellow, thick walled, fleshy, and glossy and look like conical berries at maturity, up to 5 × 5 cm in size, turbinate, and containing 6–8 mm in diameter dark brown or black color seeds [1, 19, 31]. It can be propagated by stem cutting or seeds [12].

**Figure 1:**
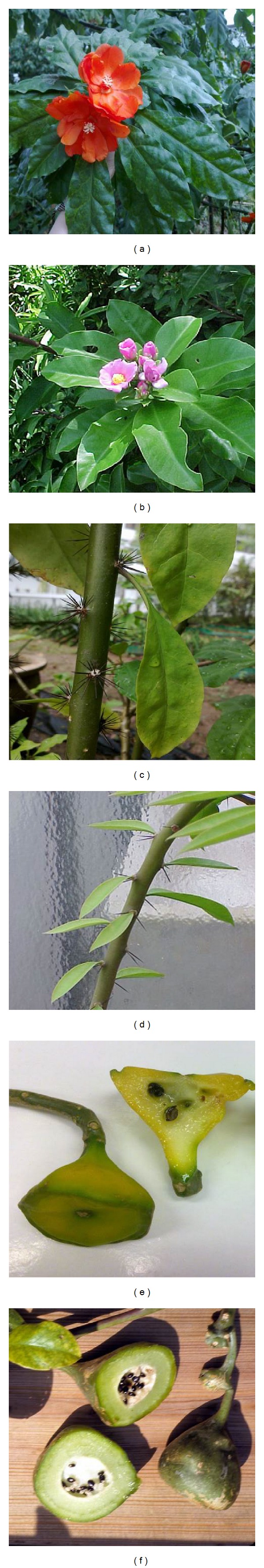
Photographs of different plant parts of* P. bleo* and* P. grandifolia.* (a) Flower of* P. bleo*, (b) lower of* P. grandifolia* [47], (c) stem and spines of* P. bleo*, (d) stem and spines of* P. grandifolia* [48], (e) ripe fruits and seeds of* P. bleo, *and (f) ripe fruits and seeds of* P. grandifolia* [49].

This species was collected by Bonpland during Humboldt's trip through the new world and was described and published by Kunth in 1823 [2]. In some older books and herbaria, it was confused with* Pereskia grandifolia* (*P. grandifolia*) [20] because both plants are vegetatively similar [31]. In addition,* P. bleo* and* P. grandifolia* are the only exceptions of* Pereskia *which grow in areas receiving considerably high annual rainfall more than 187 mm per wet month. Other* Pereskia* species grow in dry areas [3]. The two species can be distinguished by the leaves, flowers, and spines.* P. bleo* has thinner, corrugated leaves and orange-red flowers, with shorter spines compared to* P. grandifolia.* In contrast,* P. grandifolia* has thicker, uncorrugated leaves, pink to purplish-pink flowers and longer but fewer spines on the stems [11].  Figure 1 shows the photographs of different parts of* P. bleo* and* P. grandifolia*. Although they are different species, anatomical similarities in these two species support the evolution theory for cactus family [18].

### 3.2. Traditional Usage

*P. bleo *has been used for various purposes. In some areas, it is used as a food spice [1, 7]. This plant has been eaten raw as vegetables by some people in Malaysia and China or taken as a concoction brewed from fresh leaves [19, 36]. In addition, it is taken for detoxification and revitalizing the body [27, 28, 40]. Its fruit is consumed by some ethnic groups in Panama as a wild fruit [26]. The leaves of* P. bleo* have been traditionally used to treat cancer, hemorrhoid, hypertension, diabetes [32, 40], infections, gastric pain, headache, ulcer, and inflammatory conditions like rheumatism and asthma [28, 31]. Indigenous Colombians have used* P. bleo* to neutralize the effects of snakebites [33], to relax spastic muscles, and to alleviate muscle aches [29]. Apart from dietary and medicinal uses, this plant is a suitable barrier hedge because of its sharp spines, strong stem, and insect repellant properties [21]. In Central America, Kuna Indians used the crushed leaves to clarify drinking water [12]. Different methods of preparation have been reported for the plant. It is usually taken raw or as a decoction of its fresh leaves.  Table 3 shows the traditional usage and different preparation methods of* P. bleo*. To the best of our knowledge, information on the specific preparation methods for some of the indicated traditional usages is not available.

### 3.3. Phytochemistry

The leaves are the most commonly used part of* P. bleo* in traditional medicine. Hence, they have been more studied compared to the other plant parts. So far, 20 phytoconstituents have been reported in the leaves and two components from the fruit as shown in Table 4. These components include alkaloids, fatty acids, glycosides, lactones, phenolic, sterol, terpenoid, and carotenoid compounds. The major isolated component from* P. bleo *leaves is phytol [27]. In addition, Doetsch et al. [34] reported the isolation of three alkaloids, namely, 3,4-dimethoxy-*β*-phenethylamine (mescaline), 3-methoxytyramine, and tyramine, from the leaves of this plant. Vitamin E (*α*-tocopherol) [36, 37] which is well known for its antioxidant properties; 2,4-ditert-butylphenol and dihydroactinidiolide were isolated through bioassay-guided fractionation by Malek et al. [36]. Murillo et al. [26] analyzed the fruit of* P. bleo* for lutein and zeaxanthin contents. The total carotenoid content of the fruit was found to be 13.3 *μ*g/g, making* P. bleo* fruit a high carotenoid food source among the wild fruits in Panama.

The mineral content of the leaves was also investigated by using energy-dispersive X-ray microanalysis.  Table 5 shows the weight percentage of the minerals reported by Abbdewahab et al. [38]. As can be seen,* P. bleo* leaves are rich in potassium (10.16%). This is more than two times of the potassium content of tomato (4.5%), a vegetable known to be high in potassium [50]. It has been shown that a high potassium diet has an important role in lowering blood pressure [51]. Therefore, it might be one of the possible reasons for the traditional usage of* P. bleo* as a treatment for hypertension [31].

### 3.4. Pharmacological Properties

Pharmacological evaluation of plants is based on their traditional uses. Cancer is one of the main causes of mortality and morbidity. Since* P. bleo* is traditionally used to prevent and treat cancer [28, 30, 40], it has been most studied for its antiproliferative and cancer protective properties [8, 22, 28, 32, 36, 39, 40]. This is followed by investigations of its antimicrobial and antiparasitic effects* in vitro* [8, 38, 41–43, 52]. The snake venom neutralizing properties [33], antinociceptive effects [35], and toxicity [22, 31] of this plant have been evaluated through* in vivo *studies.

#### 3.4.1. Antiproliferative Properties

The effects of different* P. bleo* extracts have been reported on various cell lines* in vitro*. The crude methanol extract and its ethyl acetate fraction had significant cytotoxic effects against human nasopharyngeal epidermoid carcinoma cell line (KB) [36]. In addition, the ethyl acetate fraction was more active than the methanol extract against human colon carcinoma (HCT116) and hormone dependent breast carcinoma cell lines (MCF7) [36].  Table 6 shows the reported IC_50_ values (*μ*g/mL) for the antiproliferative effects of* P. bleo* extracts and fractions.

Gupta et al. [22] reported high tumor inhibition activity in “potato disc inhibition assay” using crown gall tumors (LC_50_ 77 ppm). Their result was accompanied by a significant DNA peak reduction in the DNA intercalation test for the methanol extract of the whole plant.

To date, no report is available on the* in vivo* antiproliferative activities of* P. bleo*.

(1)* Cytotoxic Components.* Some of the cytotoxic components in* P. bleo* have been reported.  Table 7 shows the reported IC_50_ (*μ*g/mL) values of these components in the different human cell lines. The effects of these compounds and the mixture of the isolated sterols were not as high as doxorubicin, that is, a chemotherapy drug [28]. In another study, phytol isolated from* P. bleo* leaves was found to have a significant antitumor activity against some mouse cancer cell lines [36]. 

(2)* Proposed Antiproliferative Mechanism*. The antiproliferative activity of the methanol extract of* P. bleo* against human breast carcinoma cell line (T-47D) was found to be apoptotic in nature through the activation of caspase-3 and c-myc pathways [40]. Caspase-3 and c-myc are frequently activated death proteases which catalyze the specific cleavage of many key cellular proteins. They are also essential for normal development of the tissues as well as apoptosis in the tissues and cell types [53]. Komiya et al. [54] reported the induction of apoptosis as a mechanism of action for cytotoxic activity of phytol. DNA intercalation is another proposed mechanism of antiproliferative activity for* P. bleo* [22]. However, in some studies,* P. bleo* did not show appreciable cytotoxic effect [32]. Differences in the sources of plants, extraction methods, assay methods, and cell lines can be the possible reasons for these discrepancies. On the other hand,* P. bleo* may contain some prodrugs which are metabolized to the active metabolites. Therefore, further studies are needed to better understand its antiproliferative activity.

Apart from the cytotoxic activities against cancer cell lines, crude methanol extract and its fractions (hexane, water, and ethyl acetate) did not show any cytotoxicity to the normal human fibroblast cell lines, MRC-5 [36].

#### 3.4.2. Antioxidant Activity

The adverse effects of oxidative stress on human health have become a serious issue. Oxidative stress causes production of free radicals in the body that facilitate the development of degenerative diseases such as cardiovascular diseases, cancers, neurodegenerative disorders [55], Alzheimer's, and inflammatory diseases [56]. One solution to this problem is to supplement the diet with antioxidant compounds found in natural plant sources [57]. Hence, in the literature, the antioxidant effects of* P. bleo* were evaluated using different assays as follows.

*2,2-Diphenyl-1-picrylhydrazyl Hydrate (DPPH) Assay*. The methanol, dichloromethane, ethyl acetate, and hexane extracts of* P. bleo* leaves were tested [8, 25]. The hexane extract exhibited the most effective radical scavenging activity (EC_50_ 210 *μ*g/mL) followed by the ethyl acetate extract (EC_50_ 225 *μ*g/mL). This spectrophotometric assay uses a stable radical 2,2′-diphenylpicrylhydrazyl (DPPH) as a reagent [8, 25].

*Ferric Reducing Antioxidant Potential Assay (FRAP)*. The hexane, water, and methanol extracts of* P. bleo* leaves were found to reduce Fe^3+^/ferric cyanide complex to the ferrous form. Although the reduction was statistically significant, it was not more than ascorbic acid (vitamin C) and butylated hydroxyanisole (BHA) as positive controls [25]. Hassanbaglou et al. [37] compared the antioxidant activity of the ethyl acetate extract with that of hexane, ethanol, and methanol extracts. They showed that the ethyl acetate extract had significantly higher antioxidant properties compared to the rest of the tested extracts. FRAP measures the ability of test samples to reduce ferric ion to the ferrous form of TPTZ (2,4,6-tripyridyl-s-triazine).

**β*-Carotene-Linoleic Bleaching Assay.* The ethyl acetate extract of* P. bleo* demonstrated the strongest antioxidant activity followed by the methanol extract reported by Sim et al. [25]. In this assay, the linoleate free radicals formed during the reaction are neutralized by antioxidants.

In general, although different studies used plant materials from different sources and nonsimilar extraction methods, ethyl acetate and hexane extracts appear to be the strongest antioxidant extracts from the* P. bleo* leaves [8, 25, 37]. Moreover, this antioxidant capacity is strongly associated with the total phenolic compounds and flavonoid content of the plant leaves [25, 37, 58]. The above studies suggest that* P. bleo* has antioxidant properties which can be one of the possible reasons for its traditional usage for detoxification and prevention of cancer.

#### 3.4.3. Antimicrobial Properties

*P. bleo* has been shown to possess antibacterial, antiviral, and antifungal properties* in vitro*.  Table 8 shows the effect of* P. bleo* extracts on selected bacteria and fungi. As can be seen, the methanol and hexane extracts demonstrated great antibacterial activities against* Salmonella choleraesuis* and* Pseudomonas aeruginosa*. In addition, its dichloromethane extract showed promising antibacterial effect against* Methicillin resistant Staphylococcus aureus* [8, 38]. All of the mentioned bacteria are among the main causes of nosocomial infections and they have been developing antibiotic resistance [59–61]. Therefore, the potential antibacterial activity of* P. bleo* needs to be further investigated to identify the lead(s) antibacterial component(s).

The antifungal activity of the water and methanol extract of* P. bleo* leaves against* Cladosporium cucumerinum*, a plant pathogenic fungus, has been reported [43], but they were not active against* Candida albicans*, a common human pathogen [42, 43].

The antiviral properties of the water and methanol extracts of* P. bleo* leaves were evaluated against* Herpes Simplex Virus*-I (HSV-1) and* Human Immunodeficiency Virus* (HIV) by Matsuse et al. [62]. Both of the extracts demonstrated anti-HIV activity. However, the result of this study was not promising because of the low selectivity index of 0.94. Besides, in another study by Hattori et al. [63], the same extracts did not demonstrate any antiviral activity against HSV-1. In general, the available data on the antiviral activity of* P. bleo* is neither sufficient nor conclusive. Therefore, further research needs to be carried out.

#### 3.4.4. Antiparasitic Properties

The only antiparasitic investigation on* P. bleo* was reported by Marston et al. [52]. In their study, the chloroform, methanol, and water extracts of this plant did not exert any antiparasitic activity against schistosomiasis.

#### 3.4.5. Neutralizing Snake Venom

Otero et al. [33] evaluated the neutralizing effect of the ethanol extract of* P. bleo* on hemorrhagic activity of “*Bothrops atrox* venom” in mice. This extract did not show any neutralizing effect against the tested venom.

#### 3.4.6. Antinociceptive Properties

Wahab et al. [35] evaluated the antinociceptive activity of the ethanol extract and its fractions using two* in vivo* analgesic models: peripheral formalin-induced licking and acetic acid-induced abdominal writhing. They showed that the ethanol extract, hexane fraction, dichloromethane fraction, and ethyl acetate fraction of* P. bleo* had moderate antinociceptive effects. However, no compound was identified in their study.

### 3.5. Toxicity Studies

Acute toxicity effect of the leave's extracts of* P. bleo* was evaluated by* in vitro* and* in vivo* studies. Er et al. [32] showed that the water extract may form mutagenic compound(s) upon metabolization by the liver enzymes* in vitro*. In another study by Gupta et al. [22], the methanol extract of the whole plant had moderate toxicity in brine shrimp toxicity assay (LD_50_ 77 ppm). In the only* in vivo* study by Sim et al. [31], the methanol extract did not have any toxicity effect on ICR mice (LD_50_ > 2500 mg/kg). Although animal models have around 70–80% predictability for human toxicities [64, 65], the long term toxicity and the mutagenicity of metabolites of* P. bleo* should be further investigated.

## 4. Conclusion

A comprehensive review on* Pereskia bleo* has been presented. It provides an overview of the botanical characteristics, traditional usage, phytoconstituents, pharmacological activities, and safety of* P. bleo*. The current review highlights the association between the traditional usage of the plant and the reported anticancer, antibacterial, and antinociceptive effects tested in different studies. Although* P. bleo* has been traditionally used for a variety of therapeutic and prophylactic purposes, only a few of them has been investigated. Hence, more research is warranted to further study its biological activities and chemical properties to understand its traditional usage and to develop novel therapeutics. Understanding the traditional uses, knowing the available scientific evidences, and identifying the gaps in research will allow the proper translation of promising research results into a safe and efficacious usage of herbal medicine and discovery of new therapeutics. It will also assist in setting appropriate policy and guidelines in the usage of herbal medicine.

## Figures and Tables

**Table 1:** Clades of the genus *Pereskia* [9].

Clade A	Clade B
*Pereskia aureiflora* F.Ritter *Pereskia bleo* (Kunth) DC *Pereskia guamacho* F.A.C.Weber *Pereskia lychnidiflora* DC *Pereskia marcanoi* Areces *Pereskia portulacifolia* (L.) DC *Pereskia quisqueyana* Alain *Pereskia zinniiflora* DC	*Pereskia aculeata* Mill. *Pereskia bahiensis* Gürke *Pereskia diaz-romeroana* Cárdenas *Pereskia grandifolia* Haw. *Pereskia horrida* DC *Pereskia nemorosa* Rojas Acosta *Pereskia sacharosa* Griseb. *Pereskia stenantha* F.Ritter *Pereskia weberiana* K.Schum.

**Table 2:** Scientific and common names of *P. bleo*.

Names	References
Scientific names	
*Cactus bleo* Kunth	[2, 12, 17–19]
*Pereskia bleo* (Kunth) DC	[1, 2, 12, 17, 19–21]
*Pereskia corrugata* Cutak	[17, 21]
*Pereskia panamensis* F.A.C. Weber	[2, 17]
*Rhodocactus bleo *(Kunth) F.M. Kunth	[17, 19, 21]
*Rhodocactus corrugatus* (Cutak) Backeberg	[17]
Common names	
Butarrar (Kuna Indian)	[22]
Cak Sing Cam, Qi xing zhen (Chinese)	[1, 8, 23]
Chupa, Chupa melon, Najií, Najii De Culebra, Najú de esoubas, and Bleo de chupa (Spanish)	[2, 21, 24]
Perescia	[7]
Pokok Jarum Tujuh Bilah (Malay)	[2, 25]
Rose cactus, Bleo, Chinese rose, Spinach cactus, wax rose, and orange rose cactus (English)	[1, 6, 7, 21, 24, 26]

**Table 3:** Traditional usage and methods of preparation of *P. bleo*.

Purpose	Method of preparation	References
Detoxification and prevention of cancer	Making tea by boiling the leaves and/or the fruit and then drinking it warm or cool	[27–30]

Dietary purposes and health maintenance	Eating the raw leaf, flower, and fruit	[19, 28]

Health maintenance and revitalizing the body	Making juice from the leaves and boiling in water and drinking every morning	[30]

To alleviate muscleache	Making decoction from the leaves and then using as a warm bath for muscle ache	[29]

To alleviate stomachache	Preparing “ina kuamakalet”: the inflorescence is mixed with the excrements of red ants by using a special mortar and then moistened with water. The resulted mass is moulded to oval shape objects which are dried in sun. When using the remedy, these balls are rubbed in a small container with a small amount of water.	[29]

To treat hemorrhoid, hypertension, diabetes, infections, headache, and inflammatory conditions (rheumatism and asthma)	No information is available in the literature.	[28, 31, 32]

To neutralize the effects of the snakebites	No information is available in the literature.	[33]

**Table 4:** Reported phytoconstituents in the leaves and fruits of *P. bleo*.

Plant part	Class of the constituents	Constituents	Reference
Leaves	Alkaloids	3,4-Dimethoxy-*β*-phenethylamine	[34]
		3-Methoxytyramine	[34]
		Tyramine	[34]
	Fatty acids	Methyl palmitate	[31]
		Methyl linoleate	
		Methyl *α*-linoleate	
	Flavonoid	Vitexin	[35]
	Phytosterol glycoside	*β*-Sitosterol glucoside	[35]
	Lactone	Dihydroactinidiolide	[28]
	Phenolic compounds	2,4-Ditert-butylphenol	[36]
		*α*-Tocopherol	[36, 37]
		Catechin	[37]
		Epicatechin	[37]
		Quercetin	[37]
		Myricetin	[37]
	Sterols	Campesterol	
		Stigmasterol	[28]
		*β*-Sitosterol	[36]
	Terpenoids	*β*-Carotene	[37]
		Phytol	[36]

Fruit	Carotenoids	Lutein (*β*,é-carotene-3,3′-diol)	[26, 37]
		Zeaxanthin (*β*,*β*-carotene-3,3′-diol)	

**Table 5:** Percentage (% w/w) of mineral contents in the leaves of *P. bleo* [38].

Mineral elements	Percentage weight (%)
Carbon	50.6
Oxygen	35.4
Magnesium	0.4
Phosphorus	0.4
Sulfur	1.5
Chlorine	1.2
Potassium	10.2
Aluminium	ND*
Calcium	0.3
Silicon	ND
Ferrum (Iron)	ND

*ND: not detected.

**Table 6:** IC_50_ values (*μ*g/mL) of *P. bleo* leaf extracts and fractions on different cell lines.

Cell line	Extracts and fractions (IC_50_: *μ*g/mL)					Positive control (IC_50_: *μ*g/mL)	Negative control	References
	Methanol	Water	Hexane	Dichloromethane	Ethylacetate			
4T1	>50	>50	NA	NA	NA	Cisplatin (NA)	NA	[32]
CasKi	40.5	—	89.5	NA	58	Doxorubicin (6 × 10^−3^)	NA	[36]
CEM-ss	—	NA	—	—	—	NA	VC	[8]
HT29 and HCT116	>30	NA	>30	>30	>30	NA	VC	[8]
	41.6	—	67.5	NA	22	Doxorubicin (3.6 × 10^−1^)	NA	[36]
KB	6.5	—	28	NA	4.5	Doxorubicin (1.2 × 10^−2^)	NA	[36]
MCF-7	>30	NA	>30	>30	>30	NA	VC	[8]
	39	—	25	NA	28	Doxorubicin (7.5 × 10^−2^)	NA	[36]
MRC-5	—	—	—	NA	—	Doxorubicin (5.5 × 10^−1^)	NA	[36]
NIH/3T3	≥200	≥200	NA	NA	NA	Cisplatin	NA	[32]
Saos-2	—	NA	NA	NA	NA	Cisplatin	NA	[39]
T-47D	2	NA	NA	NA	NA	DNase I	VC	[40]
V79	—	NA	NA	NA	NA	Nitracrine	VC	[22]

IC_50_: 50% of maximum cell inhibition. IC_50_ < 20 *μ*g/mL is considered active, 100 > IC_50_ > 20 *μ*g/mL is relatively active, and IC_50_ > 100 is not active [8].

(—): no activity.

4T1: mouse mammary cancer cell line; CasKi: human cervical carcinoma cell line; CEM-ss: human T-4 lymphoblastoid cell line; HT29 and HCT116: human colon carcinoma cell line; KB: human nasopharyngeal epidermoid carcinoma cell line; MCF-7: hormone dependent breast carcinoma cell line; MRC-5: normal human fibroblast cell lines; NIH/3T3: normal mouse fibroblast cell line; Saos-2: human osteosarcoma cell line; T-47D: human breast carcinoma cell line; V79: Chinese hamster lung fibroblasts.

NA: not available.

VC: vehicle control.

**Table 7:** Reported IC_50_ values (*μ*g/mL) of selected *P. bleo* phytoconstituents on human cell lines [28].

Compound	IC_50_ (*μ*g/mL) of different cell lines					
	KB	MCF7	CasKi	HCT 116	A549	MRC-5
Dihydroactinidiolide	6.7	30	40	5	97	91.3
*β*-sitosterol	>100	72	62	>100	78	>100
2,4-ditertbutylphenol	0.81	5.75	4.5	29	6	20
*α*-tocopherol	8	7.5	6	31	6	30.5
Phytol	7.1	34	18	100	31	74.1
Mixture of sterols	>100	>100	>100	>100	>100	>100
Doxorubicin	1.3 × 10^−2^	7.6 × 10^−2^	6.0 × 10^−3^	3.6 × 10^−1^	2.2 × 10^−1^	5.5 × 10^−1^

A549: human lung carcinoma cell line, CasKi: human cervical carcinoma cell Line, HCT116: human colon carcinoma cell Line, KB: human nasopharyngeal epidermoid carcinoma cell Line, MCF-7: hormone dependent breast carcinoma cell Line, MRC-5: normal human fibroblast cell Lines.

**Table 8:** Reported effects of *P. bleo* extracts on the growth of selected bacteria and fungi.

Organism	Antibacterial and antifungal effect of the extracts						Positive control	References
	Methanol	Water	Hexane	Dichloroethane	Ethyl acetate	Chloroform		
*Bacillus subtilis* ^ a^	−	NA	−	−	−	NA	Streptomycin*	[8]
	−	−	−	NA	−	NA	Gentamicin, ampicillin	[41]
	NA	NA	−	−	NA	NA	Streptomycin	[38]
*Escherichia coli* ^ b^	−	NA	NA	−	NA	NA	Streptomycin	[42]
*Escherichia coli* ^ a^	−	−	−	NA	−	NA	Gentamicin, ampicillin	[41]
*Helicobacter pylori* ^ b^	−	NA	NA	−	NA	NA	Streptomycin	[42]
*Klebsiella pneumoniae* ^ b^	−	NA	NA	−	NA	NA	Streptomycin	[42]
*Methicillin resistant Staphylococcus aureus* ^ a^	−	NA	−	+++	−	NA	Streptomycin*	[8]
	NA	NA	−	++	NA	NA	Streptomycin	[38]
*Mycobacterium smegmatis* ^ b^	−	NA	NA	−	NA	NA	Streptomycin	[42]
*Pseudomonas aeruginosa* ^ a^	++	NA	+++	+	+	NA	Streptomycin	[8]
	+	−	−	NA	+	NA	Gentamicin, ampicillin	[41]
	NA	NA	+++	+	NA	NA	Streptomycin*	[38]
*Pseudomonas aeruginosa* ^ b^	−	NA	NA	−	NA	NA	Streptomycin	[42]
*Salmonella choleraesuis* ^ a^	++	NA	+++	−	−	NA	Streptomycin*	[8]
	NA	NA	+++	−	NA	NA	Streptomycin	[38]
*Staphylococcus aureus* ^ b^	−	NA	NA	−	NA	NA	Streptomycin	[42]
*Staphylococcus aureus* ^ a^	−	−	−	NA	−	NA	Gentamicin, ampicillin	[41]
*Candida albicans* ^ c^	−	−	NA	NA	NA	−	Propiconazole, miconazole	[43]
*Candida albicans* ^ b^	−	NA	NA	−	NA	NA	Amphotericin B	[42]
*Cladosporium cucumerinum* ^ c^	+	+	NA	NA	NA	+	Propiconazole, miconazole	[43]

^a^The screening for antibacterial effect was carried out by determining the zone of inhibition using paper disc, + stands for activity between 6–9 mm, ++ stands for activity between 9–14 mm, +++ stands for activity more than 14 mm [38].

^b^(+) stands for activity at 100 *μ*g/mL for *E.  coli*, *S.  aureus*, *K.  pneumoniae*, *M.  smegmatis*, *C.  albicance*, *P.  aeruginosa *and at 12.5 *μ*g/mL for *H. pylori*, (−) stands for inactive samples.

^c^agar overlay assay and (+) stands for active extracts at 50 *μ*g/mL, (−) stands for inactive extract.

NA: not applicable as there is no report in the literature.

*Streptomycin showed 20 to 23 mm inhibition zone. The rest of the studies did not report the exact value of the inhibition for their positive controls.
